# Pancreatic juice cytology via a nasopancreatic drainage tube placed using the rendezvous technique through the accessory pancreatic duct

**DOI:** 10.1055/a-2689-3527

**Published:** 2025-09-26

**Authors:** Yoshifumi Azuma, Ryota Sagami, Hiroaki Tsuji, Takao Sato, Hidefumi Nishikiori, Kazuhiro Mizukami, Kazunari Murakami

**Affiliations:** 1 74109 Gastroenterology, Oita Red Cross Hospital, Oita, Japan; 2 13235 Gastroenterology, Oita University Faculty of Medicine, Yufu, Japan; 3 13235 Advanced Gastrointestinal Cancer Medicine, Oita University Faculty of Medicine, Yufu, Japan; 4 157533 Gastroenterology, Oita San-ai Medical Center, Oita, Japan

A 71-year-old woman visited our hospital for evaluation of asymptomatic pancreatic cysts. Magnetic resonance cholangiopancreatography (MRCP) revealed dilated branch ducts (retention cysts) surrounding a caliber change of the main pancreatic duct (MPD) in the pancreatic body ( Fig. 1 **a** ). Contrast-enhanced computed tomography (CT) demonstrated localized pancreatic parenchymal atrophy around the MPD irregularity ( Fig. 1 **b** ). Endoscopic ultrasound (EUS) also revealed retention cysts surrounding the MPD caliber change with upstream MPD dilatation ( Fig. 1 **c** ). No obvious tumor was detected on any imaging modalities. Based on these findings, isolated high grade intraepithelial neoplasia (HGIN) of the pancreas without invasive carcinoma was strongly suspected. As there was no visible mass suitable for EUS-guided tissue acquisition, pancreatic juice cytology was selected as the technique for further investigation.

**Fig. 1:**
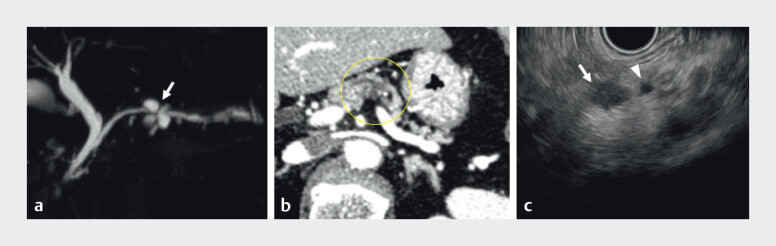
Imaging findings associated with high grade intraepithelial neoplasia of the pancreas on: **a** magnetic resonance cholangiopancreatography, showing dilated branch ducts (retention cysts) surrounding an area of main pancreatic duct (MPD) caliber change in the pancreatic body (white arrow); **b** contrast-enhanced computed tomography, showing localized pancreatic parenchymal atrophy (yellow circle) around the MPD irregularity; **c** endoscopic ultrasound, showing retention cysts (white arrow) around the MPD caliber change, with upstream MPD dilatation (white arrowhead). No obvious tumor was identified.

Endoscopic retrograde pancreatography (ERP) revealed a tortuous MPD that prevented deep guidewire insertion ( Fig. 2 **a** ; Video 1 ). A guidewire was successfully advanced from the major papilla of Vater to the minor papilla via the MPD and accessory pancreatic duct ( Fig. 2 **b** ). Pancreatic duct cannulation was achieved using the rendezvous technique through the accessory pancreatic duct via the minor papilla ( Fig. 2 **c** ), and a second guidewire was introduced deeply into the MPD ( Fig. 2 **d** ). Subsequently, an endoscopic nasopancreatic drainage (ENPD) tube was placed across the minor papilla ( Fig. 2 **e** ). Repeated cytological examinations of the fluid obtained from ENPD confirmed cytological positivity for pancreatic ductal adenocarcinoma. The patient underwent distal pancreatectomy, and the final pathological diagnosis was isolated HGIN of the pancreas without invasive carcinoma ( Fig. 3 ).

**Fig. 2:**
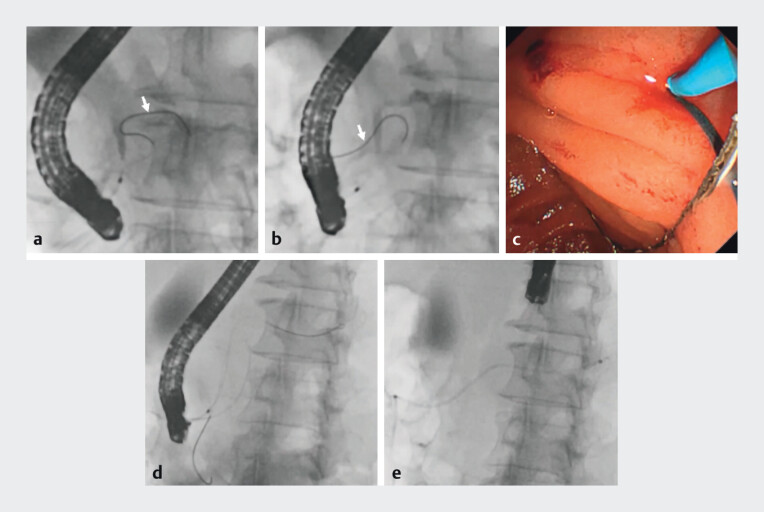
Images during nasopancreatic tube placement using the rendezvous technique through the accessory pancreatic duct showing: **a** on fluoroscopy during endoscopic retrograde pancreatography, a tortuous guidewire (white arrow) in the main pancreatic duct (MPD) preventing deeper insertion into the MPD; **b** a guidewire that was successfully introduced from the major papilla of Vater to the minor papilla via the accessory pancreatic duct; **c** cannulation of the pancreatic duct through the accessory pancreatic duct via the minor papilla using the guidewire as a lead (the rendezvous technique); **d** another guidewire that was advanced deeply into the MPD from the minor papilla; **e** an endoscopic nasopancreatic drainage tube that was successfully placed across the minor papilla.

Pancreatic juice cytology was performed on fluid obtained through an endoscopic nasopancreatic drainage tube placed using the rendezvous technique via the accessory pancreatic duct.Video 1

**Fig. 3:**
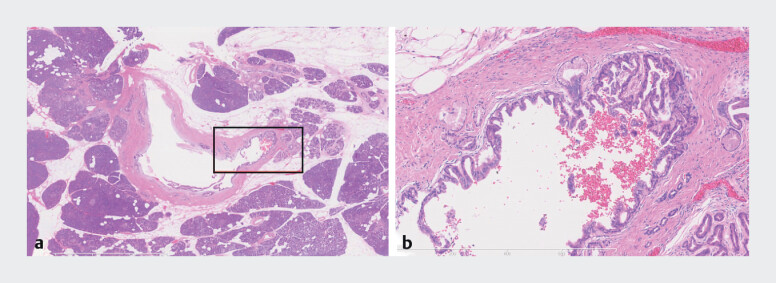
Histopathological appearance of the specimen resected at surgery showing: **a** isolated high grade intraepithelial neoplasia without invasive carcinoma (the final pathological diagnosis); **b** under moderate magnification, pancreatic epithelial neoplasia with cytoarchitectural atypia ranging from low grade to high grade – high grade dysplastic cells showed micropapillary architecture, enlarged and irregular nuclei, and loss of polarity.

Pancreatic juice cytology on fluid obtained using an ENPD tube is useful in the diagnosis of pancreatic HGIN without obvious tumor on any imaging findings 1 2 ; however, ERP can be challenging in cases of pancreas divisum or anatomical tortuosity 3 4 . This case highlights the utility of the rendezvous technique via the accessory pancreatic duct for successful ENPD tube placement in challenging ERP cases.

Endoscopy_UCTN_Code_TTT_1AR_2AD

## Notes

Endoscopy E-Videos https://eref.thieme.de/e-videos
*E-Videos* is an open access online section of the journal *Endoscopy* , reporting on interesting cases and new techniques in gastroenterological endoscopy. All papers include a high-quality video and are published with a Creative Commons CC-BY license. Endoscopy E-Videos qualify for HINARI discounts and waivers and eligibility is automatically checked during the submission process. We grant 100% waivers to articles whose corresponding authors are based in Group A countries and 50% waivers to those who are based in Group B countries as classified by Research4Life (see: https://www.research4life.org/access/eligibility/ ). This section has its own submission website at https://mc.manuscriptcentral.com/e-videos .
